# Middle cerebral artery dynamic cerebral autoregulation is impaired by infarctions in the anterior but not the posterior cerebral artery territory in patients with mild strokes

**DOI:** 10.1515/tnsci-2022-0278

**Published:** 2023-04-01

**Authors:** Manuel Bolognese, Grzegorz Karwacki, Mareike Österreich, Martin Müller, Lehel Lakatos

**Affiliations:** Department of Neurology and Neurorehabilitation, Kantonsspital Lucerne, Spitalstrasse, CH-6000 Lucerne 16, Switzerland; Section Neuroradiology, Department of Radiology, Kantonsspital Lucerne, Spitalstrasse, CH-6000 Lucerne 16, Switzerland

**Keywords:** stroke, brain connectivity network, ultrasound, transfer function, neuroscience

## Abstract

**Objective:**

The aim of this study was to ascertain whether dynamic cerebral autoregulation (CA) in the middle cerebral artery (MCA) is disturbed by cerebral infarctions outside the MCA territory.

**Methods:**

We estimated transfer function parameters gain and phase from simultaneous recordings of spontaneous oscillation in blood pressure and MCA cerebral blood flow velocity in 10 consecutive patients with isolated anterior cerebral artery (ACA) infarctions and in 22 consecutive patients with isolated posterior cerebral artery (PCA) infarctions. All ACA infarctions were in the motor, premotor, or supplementary motor cortex areas and presented with pronounced leg hemiparesis. Twenty-eight age- and sex-matched healthy subjects served as controls.

**Results:**

Compared to controls, phase was significantly reduced in the MCA ipsilateral to the lesion site and in the contralateral MCA (unaffected hemisphere) in the very low (0.02–0.07 Hz) and low (0.07–0.15 Hz) frequency ranges in the ACA infarctions but not in the PCA infarctions. Gain was reduced only in the very low frequency range in the MCA contralateral to the ACA lesion site. Systemic factors were unrelated to phase and gain results.

**Conclusion:**

Bilateral impairment of MCA dynamic CA in patients with a unilateral ACA infarction is frequent.

## 1 Introduction

Cerebral perfusion depends on various factors, such as the mean arterial pressure (MAP), cranial perfusion pressure, blood carbon dioxide and oxygen tension, blood pH value, metabolic demands, and remote influences from the brain’s functional connectivity networks (CNs) [1]. In a strict sense, the relationship between cerebral blood flow (CBF) and MAP determines cerebral autoregulation (CA); the other variables modulate this relationship, influencing overall CBF. The phenomenon of neurovascular coupling (NVC) is illustrated by cognitive tasks and arm movements that increase CBF or its velocity (CBFV, measured by transcranial Doppler [TCD] ultrasound), indicating increased perfusion [2,3,4,5,6,7]. Interestingly, such NVC studies showed additional effects in various other vascular territories, ultimately leading to the concept of brain CNs [2,3,6,7].

During an ischaemic stroke in the middle cerebral artery (MCA) territory, the dynamical aspects of CA (dCA) are frequently disturbed; interestingly, dCA is disturbed not only on the stroke-affected side but also on the unaffected side [8]. The reason for this behaviour is unclear, but systemic factors such as high blood pressure (BP) [9,10], diabetes mellitus [11], or the presence of cerebral microangiopathy [12,13] are discussed. CN considerations as a possible confounder of the dCA in the MCA have not yet been addressed. To gain a first impression on such a hypothesis, we investigated whether isolated infarctions in other supratentorial vascular territories (anterior cerebral artery [ACA]; posterior cerebral artery [PCA]) can affect dCA in the MCA. Given the body of knowledge about the motoric CNs derived from functional magnetic resonance imaging (fMRI; summarized in refs [2,3]), we hypothesize that infarctions in the motoric areas in the ACA territory should affect the dCA in the MCA, while infarctions in the PCA territory which usually do not have motor symptoms would not. Anatomically, the blood supply to the PCA is usually provided via the vertebral and the basilar arteries, and both are independent of the internal carotid artery (ICA) and the MCA. The ICA supplies both the MCA and the ACA. To avoid any contamination of our results by haemodynamically relevant ICA obstructions, we considered this possibility *a priori* and excluded patients with such relevant ICA pathologies.

## 2 Methods

The Lucerne Hospital is a large tertiary teaching hospital with a full-service stroke centre. All patients with stroke syndrome receive standardized care, first undergoing a focused clinical examination followed by multimodal cranial computed tomography. If indicated, intravenous thrombolysis and/or arterial thrombectomy immediately follows. All patients with stroke syndrome are transferred to the stroke unit for close clinical monitoring. Patients are scored on the National Institutes of Health Stroke Scale (NIHSS [14]) and modified Rankin scale [15] upon hospital admission and daily while in the stroke unit; they also undergo monitoring of MAP, heart rhythm, body temperature, blood glucose concentration, and oxygen saturation. An echocardiogram measuring the left ventricular ejection fraction, an extensive ultrasound examination with assessment of dCA, brain MRI with diffusion-weighted imaging (DWI), T2, fluid-attenuated inversion recovery, and susceptibility-weighted imaging sequences were performed within a maximum of 72 h after hospitalization. Summarizing all imaging modalities, each ischaemic event is classified according to the Trial of Org 10172 in Acute Stroke Treatment (TOAST) classification [16]. If the neurological deficit resolved within 24 h and DWI remained negative, these patients were classified as having suffered a transient ischaemic attack. Irrespective of whether the patient is symptomatic, any present cerebral microangiopathy is classified according to the Fazekas scale [17]. Infarct size is calculated by the ABC/2 method, which we evaluate manually and compare with the measurements made by automated software [18].

For this study, we recruited 10 consecutive patients with unilateral ACA infarction and 22 with PCA infarction who had received the abovementioned structured stroke care, who had temporal bone windows for TCD ultrasound examinations, and who did not have stenosis ≥50% in the extra- or intracranial arteries supplying the brain. All investigations were performed within 72 h after symptom onset. From our previously reported healthy population [19], we included 29 age- and sex-matched participants as controls and averaged the results of the bilateral MCAs as a reference value. All healthy persons were without known cardiocirculatory complains or anatomical variations.

## 3 dCA assessment

For details of the dCA assessment, see previous reports [3,19]. CBFV (MultidopX, DWL; Compumedics, Sipplingen, Germany; 2-MHz probes) and BP (Finometer Pro; Finapres Medical Systems, Amsterdam, The Netherlands) were simultaneously recorded over a minimum period of 6 min. The end-tidal pCO_2_ (ETCO_2_) was measured via nostril tubes using a capnography function built into the TCD device. The ETCO_2_ for each patient is reported as the mean ETCO_2_ over the total recording period. Cerebrovascular resistance was calculated by the mean BP over the mean CBFV. Patient recordings were performed at their stroke unit site with the patients in a resting position and the head slightly elevated. They were instructed not to make movements. Those of the controls (healthy subjects) were performed in a separate room with the same resting body position and the head slightly elevated. All recordings followed the recommendations of the CA network as much as possible [20].

**Figure 1:**
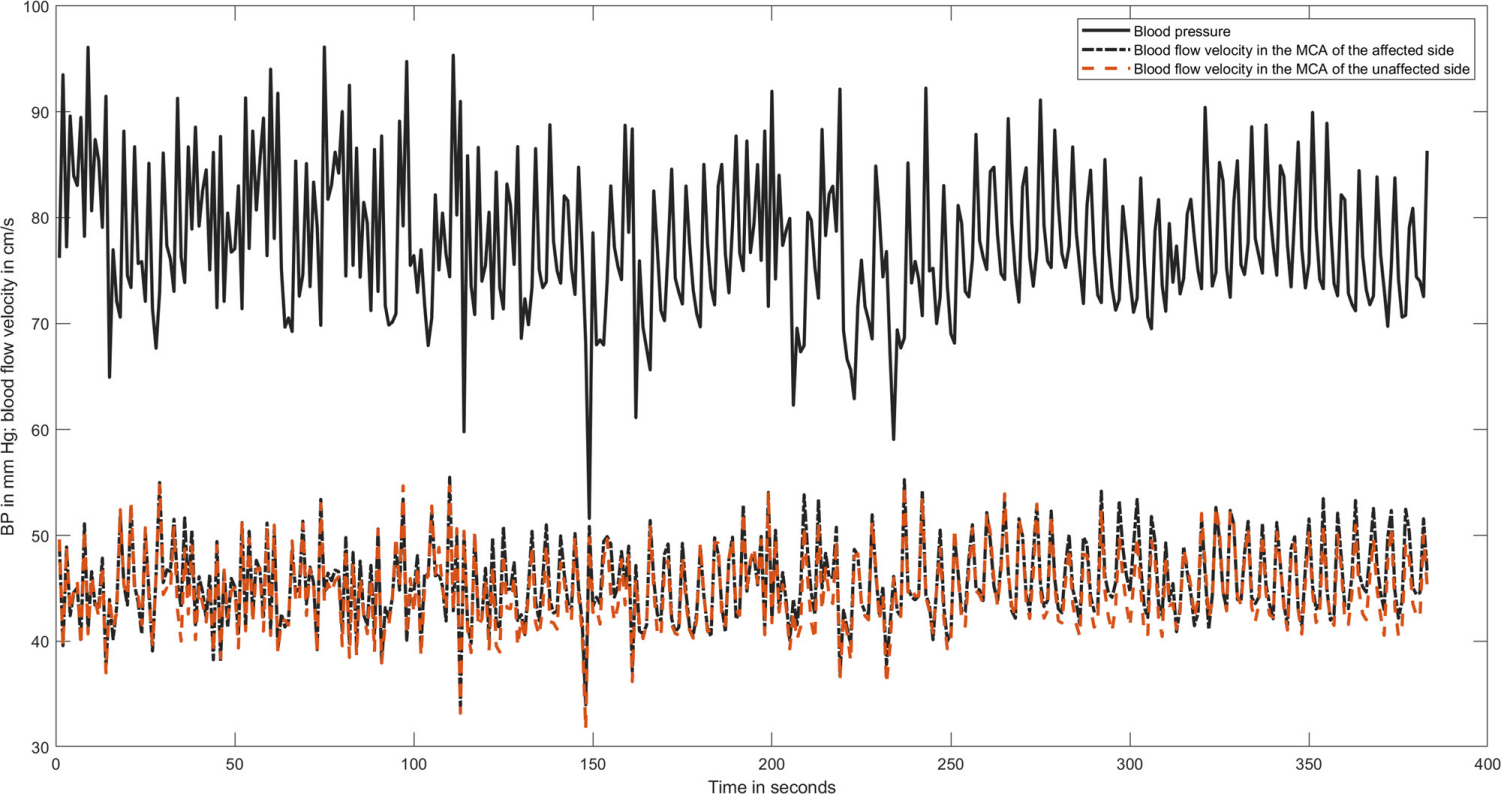
A representative recording of BP and MCA blood flow velocity in both hemispheres of a patient with an infarction in the ACA. All time series are averaged over 1 s to reduce the amount of data points. The calculated phase values were as follows: in the very low frequency 0.54 radian in the affected hemisphere and 0.64 in the unaffected one; in the low frequency phase 0.43 in both MCAs.

The data were analysed using MATLAB (2020b; MathWorks Inc., Natick, MA, USA). After visual inspection for artefacts, only artefact-free data periods of 5 min were used. Each raw data time series was averaged over 1-s intervals (one example of a patient with an ACA infarction is given in Figure 1). The transfer function estimates between BP and CBFV time series were extracted from their respective power autospectra or cross-spectra using Welch’s averaged periodogram method, with a Hanning window length of 100 s, a window overlap of 50%, and a total fast Fourier transformation data length of 300 s. Coherence, phase (in radians), and gain (in cm/s/mmHg or as %gain in %cm/s/mmHg) were calculated over a frequency range of 0.02–0.50 Hz and are reported as their respective average in the very low (VLF, 0.02–0.07 Hz), low (LF, 0.07–0.15 Hz), and high (HF, 0.16–0.5 Hz) frequency ranges. For interpretation, gain and phase usually follow an opposite direction, and an impaired dCA is indicated by a low phase and/or a high gain.

### 3.1 Statistics

MATLAB’s Statistical Toolbox was used. The Lilliefors test was used to test for the normal distribution of continuous data. Normally distributed data are reported as mean ± SD (standard deviation); nonnormally distributed data and discrete data are reported as the median [25th, 75th percentile]. To compare means, we used either a *t* test or one-way analysis of variance. To compare medians, we used the Wilcoxon rank-sum test or Kruskal‒Wallis test. To predict any parameter by regression analysis, univariate regression analysis was used. A *p* value ≤0.05 was considered indicative of statistical significance.


**Ethical approval:** The research related to human use has been complied with all the relevant national regulations, institutional policies, and in accordance with the tenets of the Helsinki Declaration, and has been approved by the authors’ institutional review board or equivalent committee. The study was approved by the Ethics Committee of Northwest and Central Switzerland (PB_2016-01719). The research is a part of the larger trial registered at ClinicalTrials.gov NCT04611672.
**Informed consent:** Informed consent has been obtained from all individuals included in this study.

## 4 Results

The overall baseline characteristics of the patients and the controls are reported in Table 1. None of the presented baseline characteristics significantly predicted the dCA parameter by regression analysis or showed significant differences between the patients with PCA or ACA infarcts. Only infarct size (see below) was different between the PCA and ACA infarctions (*p* < 0.01). The allocations of stroke-related large artery disease were in the vertebral artery system as we excluded patients with large artery disease in the carotid artery system.

**Table 1:** Patient baseline characteristics

	Patients *n* = 32	Controls *n* = 28
Age (years)	63 ± 14	63 ± 14
Women/men	11/21	8/21
Body mass index (kg/m^2^)	26 ± 4.1	
Heart rate (beats per minute)	68 ± 13	67 ± 12
MAP (mmHg)	94 ± 8	92 ± 14
Mean ETCO_2_ (mmHg)	40.0 ± 1.8	39.3 ± 4.5
Mean CBFV (cm/s)	50 ± 12	50 ± 13
Arterial hypertension	15	
Diabetes mellitus	6	
Blood glucose concentration at entry (reference range 3.8–6.4 mmol/l)	6.19 ± 1.15	
Dyslipidaemia	29	
Atrial fibrillation	3	
Left ventricular ejection fraction (%)	60 [55, 65]	
Estimated glomerular filtration rate (ml/min/1.73 m^2^)	85 [70, 91]	
Infarct volume	3.0 [0.31, 8.90]	
NIHSS at entry	2 [1, 5]	
Cerebral microangiopathy on MRI	23/1/3/5	
Fazekas grade 0/1/2/3		
TOAST	Cardioembolic 13	
	Large artery disease 5	
	Lacunar 2	
	Unknown 12	

ETCO_2_, end-tidal carbon dioxide concentration; CBFV, cerebral blood flow velocity; Ref., reference; NIHSS, National Institutes of Health Stroke Scale; MRI, magnetic resonance imaging; cerebral microangiopathy classified by the Fazekas scale (17) does not include normal brain imaging results; we classified normal brain imaging results as Fazekas grade 0 in our analysis. TOAST, Trial of Org 10172 in Acute Stroke Treatment (16).

In the patients with PCA infarctions, the median infarct volume was 4.10 ml [0.40, 12.75], with a maximum of 106 ml. Motor symptom was not present in any patient with PCA infarct. Considering the dCA parameters, there were no significant differences between the PCA infarct patients and the controls (Table 2). Thus, infarct size seems to be unrelated to dCA in the MCA.

**Table 2:** Transfer function estimates of dynamic cerebral autoregulation in the MCA in patients with acute infarctions in the PCA or ACA

Transfer function parameter	Controls (*n* = 28)	PCA infarctions (*n* = 22)		ACA infarctions (*n* = 10)	
		Affected hemisphere	Unaffected hemisphere	Affected hemisphere	Unaffected hemisphere
Coherence					
VLF	0.50 ± 0.13	0.58 ± 0.12	0.59 ± 0.11	0.57 ± 0.12	0.62 ± 0.12
LF	0.72 ± 0.15	0.68 ± 0.13	0.68 ± 0.13	0.70 ± 0.17	0.70 ± 0.16
HF	0.65 ± 0.15	0.69 ± 0.14	0.69 ± 0.16	0.67 ± 0.16	0.69 ± 0.18
Gain (cm/s/mmHg)					
VLF	0.17 ± 0.20	0.24 ± 0.16	0.28 ± 0.20	0.27 ± 0.13	0.48 ± 0.14
					*t* −3.74, df 32, *p* = 0.007
LF	0.51 ± 0.28	0.46 ± 0.28	0.45 ± 0.25	0.44 ± 0.14	0.49 ± 0.09
HF	0.57 ± 0.39	0.57 ± 0.28	0.60 ± 0.28	0.48 ± 0.15	0.59 ± 0.21
Phase (radian)					
VLF	1.09 ± 0.36	0.95 ± 0.43	0.90 ± 0.49	0.70 ± 0.30	0.42 ± 0.15
				*t* 3.00, df 36, *p* = 0.005	*t* 4.70, df 33, *p* = 0.0005
LF	0.75 ± 0.22	0.65 ± 0.27	0.68 ± 0.22	0.48 ± 0.19	0.47 ± 0.28
				*t* 3.34, df 36, *p* = 0.001	*t* 2.74, df 34, *p* = 0.009
HF	0.45 ± 0.43	0.35 ± 0.30	0.34 ± 0.32	0.11 ± 0.40	0.06 ± 0.42

PCA, posterior cerebral artery; ACA, anterior cerebral artery; VLF, very low frequency; LF, low frequency; HF, high frequency; for the respective frequency ranges, see the text. All statistical results refer to tests against the controls. *t*, *t*-statistic; df, degrees of freedom; *p*, level of significance.

**Figure 2:**
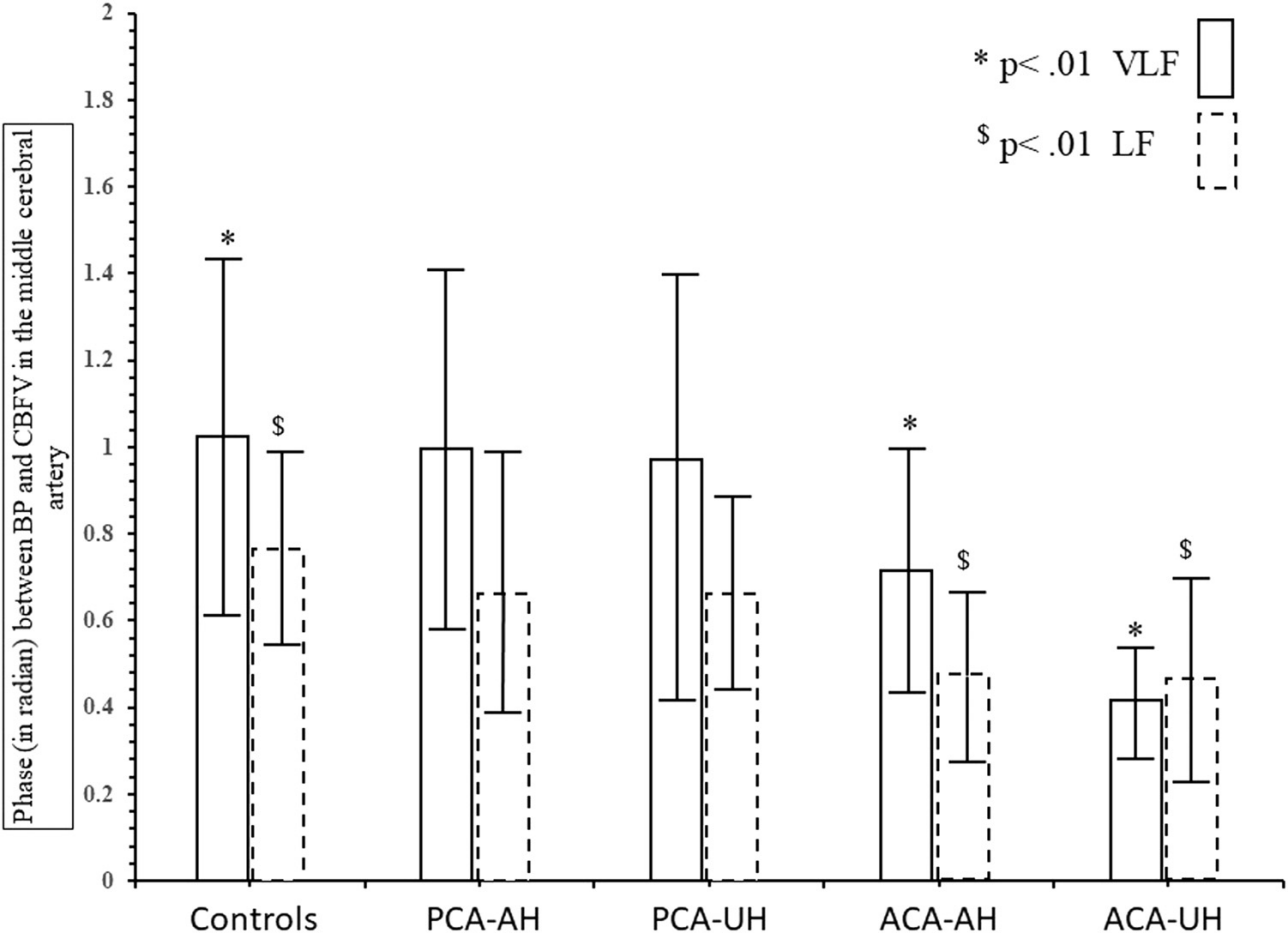
Comparison of MCA phase between controls and the patients with infarcts in the PCA or the ACA territories in the very low (VLF) and low frequency (LF) ranges.

**Figure 3:**
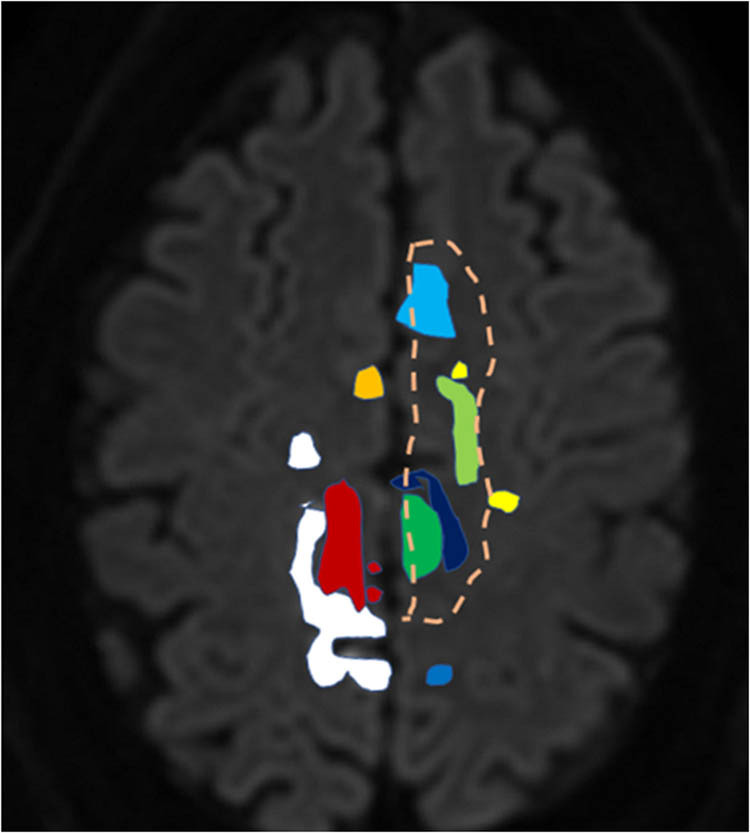
Location (*n* = 10) and horizontal extent of the infarcts in the ACA territory. Each colour represents one patient; the infarctions were allocated in the right hemisphere in three patients and in the left hemisphere in seven patients. In one patient the coloured dashed line was chosen to indicate infarct size to allow us to visualize the other infarcts which are within the boundaries of this large infarct.

In the patients with ACA infarctions, the median infarct size was 0.9 ml [0.04, 4.08], with a maximum of 29 ml. Regression analysis showed that dCA parameters were not related to infarct size. Compared to the controls (Figure 2), phase was significantly reduced in the VLF and the LF in the affected and unaffected hemispheres; the result that phase in the nonaffected side was lower than that in the affected side was mainly due to a very low outliner; phase was similarly low on both sides when this outliner was removed; therefore, our result regarding the unaffected side should not be overinterpreted given the small total number of patients. There was little difference in gain except for an increased gain in the unaffected hemisphere in the VLF range (due to the same outliner patients as in phase). Figure 3 demonstrates that nearly all infarct lesions were within the motor or premotor cortical areas. Clinically, all patients exhibited hemiparesis with the leg weaker than the arm (seven due to left-sided lesions and three due to right-sided lesions).

## 5 Discussion

Overall, PCA infarction does not affect CBF regulation in the MCA territory, while ACA infarction does with pronounced leg hemiparesis as the major motor finding. Because the main cardiovascular factors and ETCO_2_ were not different between the two stroke allocation groups, they cannot account for the different dCA findings. The sizes of infarcts in both territories were also unrelated to the dCA impairments in the MCA territory; large artery disease in the carotid artery distribution (as a common reason of dCA impairment) was excluded, and small vessel disease was rare. As Figure 1 shows, all our ACA lesions affected cortical areas relevant for motor function. We, therefore, interpret our findings as supportive for our hypothesis that motor CN contribute to MCA dCA disturbances [4,5,21,22,23].

To our knowledge, our study is the first one that describes with a bed side test that cerebral perfusion regulation in the MCA territory is affected in patients with ACA infarctions, while CBFV, as an index of CBF, is unchanged. Thus, hypothetically, dCA assessment seems more sensitive for studying even fine perfusion regulation processes and could be used for a wider approach to study motor recovery predictability [23]. Compared to MRI or other CBF-measuring techniques, the advantages of assessing dCA by TCD are its robustness and its availability as a bedside test.

This study has limitations. The environments were different between the patients and the controls. This could have influenced the results as the stroke-unit setting is noisy and could produce more noisy recordings, making the recordings more sensitive to errors [24]. However, the clear distinction between the results in the PCA infarct patients and the ACA infarct patients can be used as an argument against the relevance of the suggested noisy surroundings. The number of patients in the ACA infarct group is small, but the results are very impressive. The mechanism behind the resulting phase decrease is not directly derivable from our data, but the fact that gain as an index of the vascular tone remained widely unchanged favours a metabolic stimulus.

## References

[1] Claassen JAHR, Thijssen DHJ, Panerai RB, Faraci FM. Regulation of cerebral blood flow in humans: physiology and clinical implications of autoregulation. Physiol Rev. 2021;101:1487–559. 10.1152/physrev.00022.2020 PMC8576366 33769101

[2] Raimondo L, Oliveira ĹAF, Heij J, Priovoulos N, Kundu P, Leoni RF, et al. Advances in resting state fMRI acquisitions for functional connectomics. Neuroimage. 2021;243:118503. 10.1016/j.neuroimage.2021.118503. PMID: 34479041. 34479041

[3] Gaberova K, Pacheva I, Ivanov I. Task-related fMRI in hemiplegic cerebral palsy-A systematic review. J Eval Clin Pract. 2018;24:839–50. 10.1111/jep.12929. PMID: 29700896. 29700896

[4] Müller M, Österreich M. Cerebral microcirculatory blood flow dynamics during rest and a continuous motor task. Front Physiol. 2019;10:1355. 10.3389/fphys.2019.01355. PMID: 31708802; PMCID: PMC6821676. PMC6821676 31708802

[5] Müller M, Österreich M. Cerebrovascular dynamics during continuous motor task. Physiol Res. 2019;68:997–1004. 10.33549/physiolres.934147. PMID: 31647292. 31647292

[6] Khalaf A, Sybeldon M, Sejdic E, Akcakaya M. A brain-computer interface based on functional transcranial Doppler ultrasound using wavelet transform and support vector machines. J Neurosci Methods. 2018;293:174–82. 10.1016/j.jneumeth.2017.10.003. PMID: 29017899. 29017899

[7] Bleton H, Perera S, Sejdić E. Cognitive tasks and cerebral blood flow through anterior cerebral arteries: A study via functional transcranial Doppler ultrasound recordings. BMC Med Imaging. 2016;16:22. 10.1186/s12880-016-0125-0. PMID: 26969112; PMCID: PMC4788871. PMC4788871 26969112

[8] Nogueira RC, Aries M, Minhas JS, H Petersen N, Xiong L, Kainerstorfer JM, et al. Review of studies on dynamic cerebral autoregulation in the acute phase of stroke and the relationship with clinical outcome. J Cereb Blood Flow Metab. 2022;42:430–53. 10.1177/0271678X211045222. PMID: 34515547; PMCID: PMC8985432. PMC8985432 34515547

[9] Serrador JM, Sorond FA, Vyas M, Gagnon M, Iloputaife ID, Lipsitz LA. Cerebral pressure-flow relations in hypertensive elderly humans: Transfer gain in different frequency domains. J Appl Physiol (1985). 2005;98:151–9. 10.1152/japplphysiol.00471.2004 15361517

[10] Zhang R, Witkowski S, Fu Q, Claassen JA, Levine BD. Cerebral hemodynamics after short- and long-term reduction in blood pressure in mild and moderate hypertension. Hypertension. 2007;49:1149–55. 10.1161/HYPERTENSIONAHA.106.084939 17353511

[11] Vianna LC, Deo SH, Jensen AK, Holwerda SW, Zimmerman MC, Fadel PJ. Impaired dynamic cerebral autoregulation at rest and during isometric exercise in type 2 diabetes patients. Am J Physiol Heart Circ Physiol. 2015;308:H681–7. 10.1152/ajpheart.00343.2014 PMC4385994 25599569

[12] Purkayastha S, Fadar O, Mehregan A, Salat DH, Moscufo N, Meier DS, et al. Impaired cerebrovascular hemodynamics are associated with cerebral white matter damage. J Cereb Blood Flow Metab. 2014;34:228–34. 10.1038/jcbfm.2013.180 PMC3915198 24129749

[13] Liu Z, Ma H, Guo ZN, Wang L, Qu Y, Fan L, et al. Impaired dynamic cerebral autoregulation is associated with the severity of neuroimaging features of cerebral small vessel disease. CNS Neurosci Ther. 2022;28:298–306. 10.1111/cns.13778 PMC8739047 34894087

[14] Lyden P, Brott T, Tilley B, Welch KM, Mascha EJ, Levine S, et al. Improved reliability of the NIH Stroke Scale using video training. NINDS TPA Stroke Study Group. Stroke. 1994;25:2220–6. 10.1161/01.str.25.11.2220 7974549

[15] Van Swieten JC, Koudstaal PJ, Visser MC, Schouten HJ, van Gijn J. Interobserver agreement for the assessment of handicap in stroke patients. Stroke. 1988;19:604–7. 10.1161/01.str.19.5.604 3363593

[16] Adams HP, Jr, Bendixen BH, Kappelle LJ, Biller J, Love BB, Gordon DL, et al. Classification of subtype of acute ischemic stroke. Definitions for use in a multicenter clinical trial. TOAST. Trial of Org 10172 in Acute Stroke Treatment. Stroke. 1993;24:35–41. 10.1161/01.str.24.1.35 7678184

[17] Fazekas F, Barkhof F, Wahlund LO, Pantoni L, Erkinjuntti T, Scheltens P, et al. CT and MRI rating of white matter lesions. Cerebrovasc Dis. 2002;13(Suppl 2):31–6. 10.1159/000049147 11901240

[18] Lakatos L, Bolognese M, Müller M, Österreich M, von Hessling A. Automated supra- and infratentorial brain infarct volume estimation on diffusion weighted imaging using the RAPID software. Front Neurol. 2022;13:907151. 10.3389/fneur.2022.907151. PMID: 35873774; PMCID: PMC9304979. PMC9304979 35873774

[19] Müller M, Österreich M, von Hessling A, Smith RS. Incomplete recovery of cerebral blood flow dynamics in sufficiently treated high blood pressure. J Hypertens. 2019;37:372–9. 10.1097/HJH.0000000000001854 PMC6365247 29995701

[20] Panerai RB, Brassard P, Burma JS, Castro P, Claassen JA, van Lieshout JJ, et al. Cerebrovascular Research Network (CARNet). Transfer function analysis of dynamic cerebral autoregulation: A CARNet white paper 2022 update. J Cereb Blood Flow Metab. 2023;43(1):3–25. 10.1177/0271678X221119760. PMID: 35962478. PMC9875346 35962478

[21] Havsteen I, Madsen KH, Christensen H, Christensen A, Siebner HR. Diagnostic approach to functional recovery: Functional magnetic resonance imaging after stroke. Front Neurol Neurosci. 2013;32:9–25. 10.1159/000346408. PMID: 23859959. 23859959

[22] Michely J, Volz LJ, Hoffstaedter F, Tittgemeyer M, Eickhoff SB, Fink GR, et al. Network connectivity of motor control in the ageing brain. Neuroimage Clin. 2018;18:443–55. 10.1016/j.nicl.2018.02.001. PMID: 29552486; PMCID: PMC5852391. PMC5852391 29552486

[23] Chi NF, Ku HL, Chen DY, Tseng YC, Chen CJ, Lin YC, et al. Cerebral motor functional connectivity at the acute stage: An outcome predictor of ischemic stroke. Sci Rep. 2018;8:16803. 10.1038/s41598-018-35192-y. PMID: 30429535; PMCID: PMC6235876. PMC6235876 30429535

[24] Liu J, Simpson DM, Panerai RB. Point-Counterpoint: Transfer function analysis of dynamic cerebral autoregulation: To band or not to band? J Cereb Blood Flow Metab. 2022. 10.1177/0271678X221098448. PMC10414009 35510667

